# Factors Predicting the Surgical Risk of Osteoporotic Vertebral Compression Fractures

**DOI:** 10.3390/jcm8040501

**Published:** 2019-04-12

**Authors:** Fu-Cheng Kao, Yu-Jui Huang, Ping-Yeh Chiu, Ming-Kai Hsieh, Tsung-Ting Tsai

**Affiliations:** Department of Orthopaedic Surgery, Spine Section, Bone and Joint Research Center, Chang Gung Memorial Hospital and Chang Gung University College of Medicine, Taoyuan 333, Taiwan; afogi73@gmail.com (F.-C.K.); rr821028@gmail.com (Y.-J.H.); jobphage@gmail.com (P.-Y.C.); stock-best@yahoo.com.tw (M.-K.H.)

**Keywords:** vertebral compression fracture, osteoporosis, sagittal alignment, spinopelvic parameters

## Abstract

The aim of our study was to investigate the association between global spinal alignment, spinopelvic parameters, and outcomes of osteoporotic vertebral compression fractures (OVCF). Patients with vertebral compression fractures seen at our hospital between October 2017 and November of 2018 with a bone mineral density (BMD) T-score < −2.5 were recruited for the study. Surgical intervention was performed after eight weeks of conservative treatment depending on clinical symptoms and the willingness of patients. Spinopelvic and sagittal alignment parameters were compared between patients who had surgery and those that did not. Seventy-nine patients were included in the study. Twenty-five patients (31.6%, mean age: 73.28 ± 9.78 years) received surgery, and 54 (68.3%, mean age: 73 ± 8.58 years) conservative treatment only. Pelvic tilt, pelvic incidence, and local kyphotic angle were statistically different between the groups (all *p* < 0.05). A sagittal vertical axis ≥ 50 mm, distance between the C7 plumb line and the center of the fractured vertebra (DSVA) ≥ 60 mm, pelvic incidence outside of the range of 44 to 62°), and pelvic tilt ≥ 27° were associted with the need for surgical intervention. Measurement of spinopelvic parameters can predict the need for surgery in patients with OVCF.

## 1. Introduction

OVCFs occur spontaneously, or, more commonly, occur as a result of minimal trauma from day-to-day activities, such as bending forward, twisting, lifting objects, and even sitting from a standing position onto a low chair [1]. They are a common cause of morbidity, and are the most common fragility fractures in postmenopausal women. Men older than 65 years are also at increased risk of vertebral compression fractures, but their risk is less than that of women of the same age [2]. The initial treatment of OVCFs includes medication for pain control, rehabilitation, and early mobilization to prevent complications such as further bone loss, general deconditioning, the development of pressure sores, and pulmonary compromise [3,4]. Most patients with OVCF after nonsurgical treatment have minor or no symptoms, with few or no functional limitations [5,6,7]. However, intractable back pain and persistent dysfunction may still be present in some patients after 4–6 weeks of conservative management. These patients are the candidates for percutaneous vertebral augmentation, such as vertebroplasty and kyphoplasty [8]. Early surgical intervention can improve the prognosis and prevent complications related to prolonged inactivity, such as progressive muscle weakness, crowding of internal organs, respiratory problems, emotional problems, and even mortality [9]. However, it is very difficult to predict which patients will have a poor response to conservative management. 

Ideal spinal alignment of the human body maintains the center of gravity within the cone of economy, a concept which was first introduced by Jean Dubousset in 1994 [10]. This ideal alignment requires precise interaction of spinal and spinopelvic parameters. Furthermore, appropriate spinopelvic alignment is vital for energy-efficient posture and ambulation [11]. Trunk imbalance leads to considerable muscular demand, fatigue, and back pain, as well as disability [12]. Spinopelvic parameters, such as sagittal vertical axis (SVA), pelvic incidence (PI), sacral slope (SS), and lumbar lordosis (LL), can assist in assessing spinal alignment. Some studies have shown that spinopelvic parameters affect the union of OVCFs, and the occurrence of complications such as adjacent vertebral fractures after surgical intervention [13,14]. Therefore, spinopelvic parameters and global spinal alignment might have an effect of the outcome of OVCFs, and influence the response to nonsurgical treatment. 

The aim of this study was to investigate the association between global spinal alignment, spinopelvic parameters, and outcomes of OVCFs in order to predict the response to nonsurgical treatment of OVCFs.

## 2. Experimental Section

Patients with vertebral compression fractures seen at our hospital between October 2017 and November of 2018 with a bone mineral density (BMD) T-score < −2.5 were recruited for this prospective study. Exclusion criteria: (1) Incomplete imaging evaluation and clinical assessment; (2) spinal fixation with instrumentation was required due to complications such as a tumor, infection, or burst fracture; (3) neurological deficits due to the spinal fracture; (4) the use of a medication (e.g., steroids) or other underlying disease (e.g., end-stage renal disease) that increases the risk of a spinal fracture; (5) follow-up of <12 months; and (6) T-score > −2.5 (to exclude high energy trauma). This prospective cross-sectional cohort study was approved by the Institutional Ethical Committee, and all patients provided written informed consent. 

At the first visit, clinical and imaging assessments including thoracolumbar radiography, whole-spine lateral plain films, BMD, magnetic resonance imaging (MRI), visual analog scale (VAS), and Oswestry disability index (ODI) functional scores were performed. The diagnosis was established by the clinical examination and radiographic evaluation, and confirmed by the evaluation of bony edema and signal change on T1-weighted MRI. All patients were given same dosage and duration of oral analgesic and muscle relaxant (ultracet and methocarbamol) for pain control. At the second evaluation, performed 2 weeks after the first visit, patients with OVCFs, which were confirmed by the results of a BMD T-score < −2.5 and MRI, were enrolled in this prospective cross-sectional cohort study. All enrolled patients were begun on medications to treat osteoporosis. 

Eight weeks after the initial evaluation, a third evaluation was performed that included ODI and VAS scores, spine dynamic (flexion/extension) radiography, and whole-spine lateral plain films. At 8 weeks, the success or failure of conservative treatment was evaluated, and patients who failed conservative treatment were offered surgery (vertebroplasty or kyphoplasty). Failure of conservative treatment was defined as (1) intractable back pain with a VAS pain score > 6, (2) painful spine instability (spine dynamic radiography showing a vacuum phenomenon with severe axial back pain), and (3) patient requested to be seen before 8 weeks due to severe pain. 

**Figure 1:**
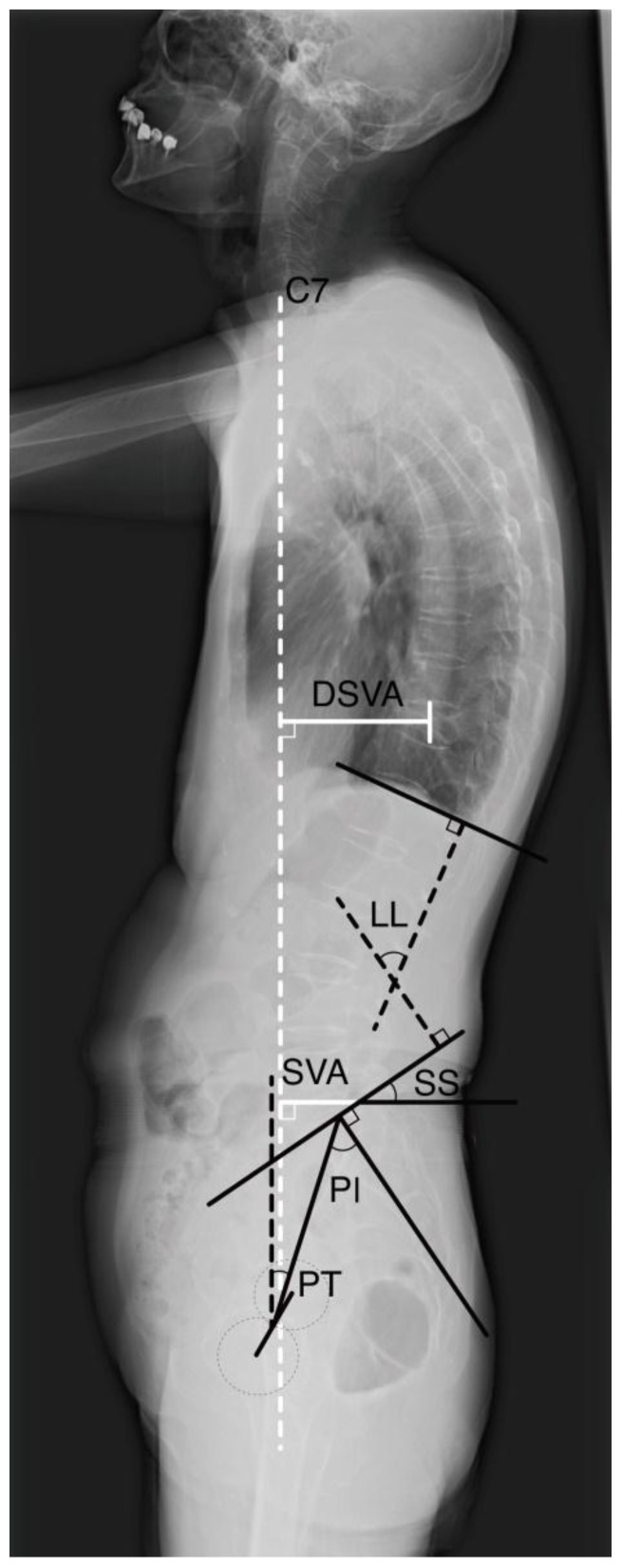
Measurement of spinopelvic parameters, sagittal vertical axis (SVA) and center of the fractured vertebra (DSVA). Owing to the nature and deviation of the spine lateral plain films, we defined the center of the femoral head used in the measurement of this study as the midpoint between femoral head of bilateral side. PI was defined as the angle between the vertical line of the sacral plateau and the line drawn from the mid-sacral plateau to the center of the femoral head. PT was defined as the angle between a line drawn from the mid-sacral plateau to the center of the femoral head and plumb line of the center of the femoral head. SS was defined as the angle between the sacral plateau and a horizontal line. Following Cobb’s method, LL was defined as the angle between the superior endplates of the L1 and S1 vertebrae. SVA was defined as the distance from the posterosuperior corner of S1 to the C7 plumb line. DSVA was defined as the distance between the C7 plumb line and the center of the fractured vertebra.

Local kyphotic angle of the fractured vertebra, pelvic incidence (PI), pelvic tilt (PT), sacral slope (SS), lumbar lordosis (LL), sagittal vertical axis (SVA), and distance between the C7 plumb line and the center of the fractured vertebra (DSVA) were measured on whole-spine lateral plain films. Local kyphotic angle was defined as the angle between the upper and lower endplate of the fractured vertebrae. Owing to the nature and deviation of spine lateral plain films, we defined the center of the femoral head as the midpoint between the femoral head of the bilateral side. PI, the morphological parameter that plays a key role in the regulation of positional pelvic and spinal factors, was measured as the angle between the vertical line of the sacral plateau and a line drawn from the mid-sacral plateau to the center of the femoral head. The angle between a line drawn from the mid-sacral plateau to the center of the femoral head and the plumb line of the center of the femoral head was defined as PT. SS was defined as the angle between the line of the sacral plateau and horizontal line. Following Cobb’s method, LL was defined as the angle between the superior endplates of L1 and the S1 vertebrae. SVA was defined as the distance from the posterosuperior corner of S1 to the C7 plumb line (Figure 1). 

All clinical and radiographic data was independently reviewed by one orthopedic spine surgeon. Data were expressed as mean ± standard deviation (SD). The significance of differences between the surgery group and conservative treatment group were evaluated using the chi-squared test, Fisher’s exact test, and Student’s *t*-test. Values of *p* < 0.05 were considered to indicate statistical significance. Univariate logistic regression analysis was used to evaluate cut-off values of spinopelvic parameters and sagittal alignment for the risk of surgical treatment. Variables significant in univariate regression analysis were entered as independent variables in multivariate logistic regression analysis, to establish a regression model. Finally, receiver operating characteristic (ROC) curve analysis [15,16] and the sensitivity, specificity, and accuracy of factors predictive of the need for surgery were calculated. All data management and statistical analyses were performed using SAS software (version 9.4; SAS Institute, Cary, NC, USA) and SPSS version 24.0 for Windows by a single professional statistician.

## 3. Results

### 3.1. Patients

**Figure 2:**
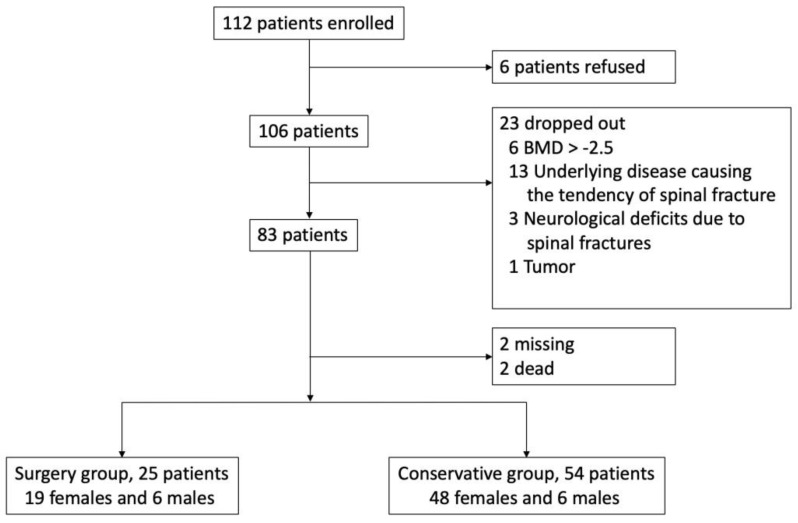
During the study period, 112 patients were seen at our hospital due to compression fractures. Six patients declined to participate in the study, and 106 of the 112 patients were enrolled in this prospective cross-section cohort study. Twenty-three patients were excluded; 6 patients had a T-score > −2.5, 13 had an underlying disease that increased the risk of a spinal fracture, 3 had neurological deficits after fractures, and one was found to have spinal metastasis. An additional 4 patients were also excluded; 2 were lost to follow-up and 2 died during the follow-up period.

From October 2017 to November 2018, 112 patients were seen at our hospital due to vertebral compression fractures. Ultimately, 79 patients with more than 12 months of follow-up were included in the study. The reasons for patient exclusion are shown in Figure 2. Of the 79 patients, 25 (31.6%, 19 females and six males) received surgery, and 54 (68.3%, 48 females and six males) received conservative management only. The mean age of the surgery group was 73.28 ± 9.78 years, with mean BMD of −3.28 ± 2.29 and a mean body mass index (BMI) of 24.33 ± 5.5 kg/m^2^. The mean age of the conservative group was 73 ± 8.58 years, with a mean BMD of −2.71 ± 2.09 and mean BMI of 24.85 ± 3.28 kg/m^2^. The demographic and clinical characteristics of the two groups are summarized in Table 1. Patients in the surgery group had significantly higher vacuum clefts of fractured vertebrae, preoperative VAS pain scores, and ODI scores at eight weeks than the conservative group.

### 3.2. Spinopelvic Parameters and Sagittal Alignment

Results of the analysis of spinopelvic parameters and sagittal alignment at the first visit between the surgery and conservative treatment groups are shown in Table 2. PI was significantly higher (*p* = 0.021) in the surgery group (62.68 ± 15.35°) than in the conservative group (56.76 ± 10.48°). In the analysis of spinopelvic parameters and sagittal alignment correlated to the need for surgical treatment; PT (univariate logistic regression *p* = 0.008) and local kyphotic angle (univariate logistic regression *p* = 0.027) had statistical significance. There was no significant difference between the surgery and conservative management groups with respect to SVA (71.89 ± 39.89 mm vs. 53.73 ± 49.6 mm, respectively), DSVA (64.09 ± 44.09 mm vs. 49.27 ± 48.14 mm, respectively), SS (33.28 ± 8.69° vs. 34.35 ± 9.61°, respectively), and LL (35.8 ± 15.74° vs. 34.85 ± 21.54°, respectively). 

### 3.3. Prediction of Risk of Surgical Intervention

**Figure 3:**
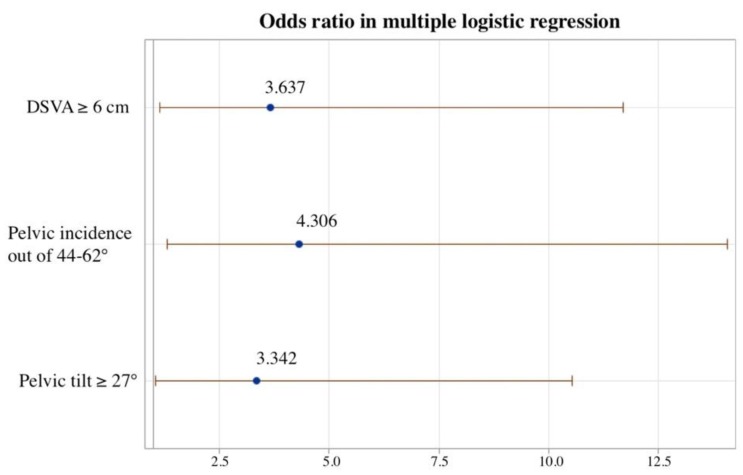
Plot for odds ratio in multivariate logistic regression. Multivariate logistic regression analysis found that PT > 27°, PI outside the range of 44° to 62°, and DSVA ≥ 6 cm (OR = 3.342, 4.306 and 3.637, respectively), were associated with failure of conservative treatment and the need for surgery.

**Figure 4:**
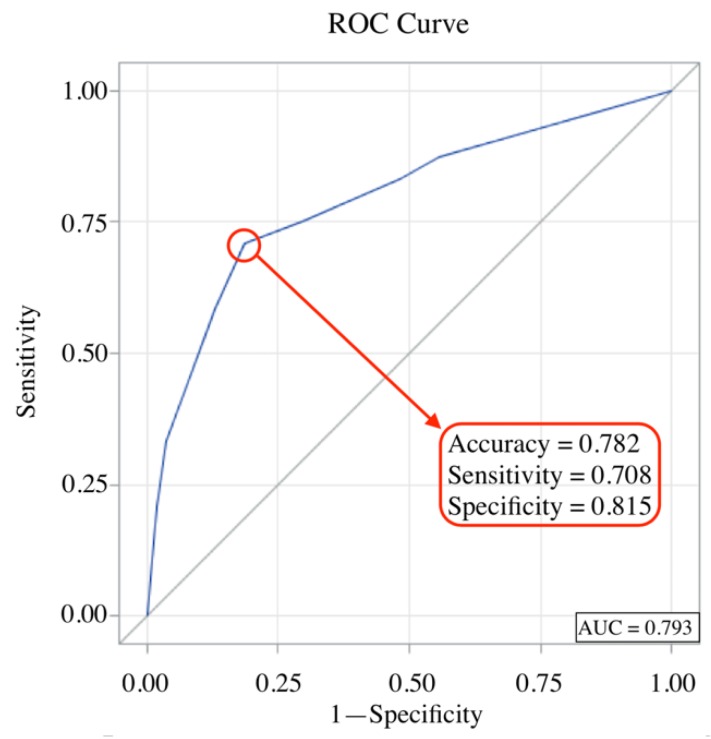
Sensitivity, specificity, and accuracy of DSVA, PI, and PT for the need for surgical intervention.

To estimate the risk of surgical intervention from spinopelvic parameters and sagittal alignment, cut-off values were calculated by univariate logistic regression analysis. Results of analysis showed that an SVA ≥ 5 cm, DSVA ≥ 6 cm, PT ≥ 27°, and PI outside of the range of 44 to 62° were associated with failure of conservative management and the need for surgical treatment (Table 3). Multivariate logistic regression analysis showed that three variables—PT ≥ 27°, DSVA ≥ 6 cm, and PI outside of the range of 44 to 62°—were associated with failure of conservative treatment with odds ratios (ORs) of 3.342, 3.673 and 4.306, respectively (Figure 3). The accuracy of these three variables for predicting the need for surgery was 0.782 in receiver operating characteristic (ROC) curve analysis (Figure 4).

## 4. Discussion

Sagittal balance of the spine has become a clinical important factor for the assessment of the spine. Spinopelvic parameters LL, PI, PT, and SS are indirect methods for checking spine sagittal balance, and are important morphological and positional indices for determining the sagittal curvature of the spine. However, the initial evaluation of sagittal balance is based on the sagittal vertical axis (SVA), which can represent the state of compensation or decompensation of overall sagittal alignment [17]. The spine and pelvis can be considered a linear chain from the head to the legs in the sagittal plane. SVA can be used to determine spinal sagittal balance on whole-spine lateral radiographs, and has a normal range. Endo et al. [18] described a series of 52 healthy adults without any symptoms or history of spinal disease or back pain, and SVA ranged from −49 mm to 70 mm. Studies have shown that SVA compensates to maintain a gravity line (center of force) while standing [19], and well-adjusted alignments are critical for balance control [20]. To maintain an upright posture, the spine and pelvis distribute weight harmoniously, and correspond with each other. For patients with sagittal spinal malalignment, compensatory mechanisms to maintain upright posture include thoracic hypokyphosis, lumbar hyperlordosis, pelvic retroversion, knee flexion, and hip extension [21]. However, in elderly individuals lumbar lordosis is often reduced, and thoracic kyphosis increases with aging [22,23]. Once these mechanisms can no longer maintain an erect posture and adjust for sagittal deformities, decompensation of the spine frequently results in poor balance control, back pain, vertebral compression fractures, and poor quality of life [20,21]. Spinopelvic imbalance may reflect the severity of underlying conditions such as osteoporosis, multiple compression fractures, and degree of pain. Lee et al. [24] reported that the SVA was significantly greater in patients with osteoporosis than normal controls. Another study showed that a greater SVA was a risk factor for delayed union of OVCFs [14]. Sagittal balance has a great influence on the symptoms and prognosis of OVCFs, and also affects surgical decision making and surgical planning [25]. In our study, SVA was greater in patients requiring surgical intervention for pain relief (71.89 ± 39.89 mm), which indicated that positive sagittal imbalance would cause severe intractable pain and fracture nonunion after a vertebral compression fracture. Univariate logistic regression analysis showed that SVA ≥ 50 mm was associated with an increased risk of the need for surgical intervention (OR = 3.09, *p* = 0.027) (Table 3). 

Another spinal–pelvic parameter that represents overall alignment is DSVA, which was first described Iwata et al. in 2017 [14], and is defined as the distance between the C7 plumb line and the center of the fractured vertebral body. DSVA was introduced as a parameter that is associated with bending moment at the fracture site. In patients with OVCFs, a larger DSVA usually suggested a hyperkyphotic posture and more flexion at the fracture site, and may interfere with bone union. In a previous study, a DSVA ≥ 50 mm was reported to be a significant risk factor affecting the union status of fresh thoracolumbar compression fractures in 48 patients [14]. However, the study was a prospective investigation and only enrolled nonoperative patients, which may have caused selection bias due to ignoring patients who required surgical intervention. In our study, DSVA was also higher in patients who failed conservative treatment (64.09 ± 44.09 mm vs. 49.27 ± 48.14 mm). We also found a DSVA cut-off point of 60 mm predicted failure of conservative treatment in univariate logistic repression analysis (OR = 3.31, *p* = 0.018) (Table 3). Multivariate logistic repression analysis showed DSVA was one of three important variables predicting the need for surgical intervention (Figure 3, OR = 3.673). Based on these findings, SVA and DSVA could be used as potent prediction factors for the need of surgical intervention in patients with osteoporotic compression fractures. 

A well-balanced erect position can be evaluated not only through measuring SVA, but also by evaluating pelvic parameters, such as pelvic incidence (PI), pelvic tilt (PT), lumbar lordosis (LL), and sacral slope (SS) [26]. When evaluating and treating spinal disorders, the spinopelvic balance is of primary importance. A prior study reported standard reference values for PI, PT, LL, and SS of 53° ± 9°, 12° ± 6°, 66° ± 9°, and 41° ± 7°, respectively [27]. To estimate the balance and compensation of the pelvis, PT is considered an important index in pathologic conditions. For patients with sagittal imbalance, retroversion of pelvis occurs for compensation, and PT is increased to maintain an upright position. Kim et al. [28] suggested that vertebral fractures could occur in patients with sagittal imbalance and a greater than average PT. The relationship of PT and surgical risk among patients with vertebral compression fractures were further analyzed in our study. Patients with a greater PT had a poor response to conservative treatment and required vertebroplasty or kyphoplasty to relieve intractable axial back pain (*p* = 0.008, Table 2). The cut-off point for PT was set to 27°, and univariate logistic regression analysis showed that patients with PT ≥ 27° were associated with increased risk of the need for surgery as compared to them with PT < 27° (OR = 5.61, *p* = 0.001, Table 3). In the multivariate logistic repression model, PT ≥ 27° was also one of three factors predicting the need for surgery (OR = 3.342, Figure 3). 

PI, which is an independent morphological constant, determines the relation between pelvic angle and sagittal spinal alignment [29]. Some authors have reported that PI is constant in adulthood, and tends to change slightly during growth [30,31]. PI is believed to remain unchanged regardless of posture or pelvic position, and there is no significant difference between sexes or at different ages [31,32]. However, decompensation of PI is common cause of several spinal diseases, such as disc herniation, degenerative spondylolisthesis, and vertebral compression fractures [29]. Numerous studies have stressed the correlation between PI and OVCFs. Lee et.al [24] investigated a series of 124 osteoporotic patients, and found PI was much higher in sagittal imbalance and osteoporosis patients, as compared to normal controls. Dai et al. [33] studied 1044 postmenopausal women, and found that lower PI was associated with a higher risk of vertebral compression fractures. Therefore, it appears that deviation of PI from the normal range increase the risk for OVCFs. In our study, PI of the surgery group was significantly higher than that of the conservative group (*p* = 0.021, Table 2). Although univariate logistic regression analysis showed no correlation between PI increase and the risk of the need for surgical intervention, the risk of surgical intervention was the lowest when PI was in the range of 40° to 60°. The risk for surgery had a U-shaped distribution on Loess plot, and increased steadily when PI was above or below the range of 40–60°. The normal range of PI in adults with no history of spine pathology is accepted to be between 44° and 62° degrees [27,34]. PI has also been used to evaluate the influence of spinal sagittal balance in spinal diseases by Barrey et al. [29]. As such, it is reasonable that we found a significant difference of abnormal PI (outside of the range of 44° to 62°) between the surgery and conservative groups. Multivariate logistic regression analysis also confirmed that PI above or below the normal range was a risk factor for the need for surgical intervention (OR = 4.306, Figure 3). 

There is a belief that spinal–pelvic parameters are correlated with each other to achieve a balanced spine alignment in the sagittal plane. The spine and pelvis are interdependent, and their relation is critical to proper posture. PI, PT, LL, and SS are significantly related, and regulate each other [27,30]. To achieve a stable upright position, sagittal balance requires several adjustments. For example, LL might be lost with aging, and cause anterior tilting of the trunk. An increase of PT and retroversion of pelvis subsequently occur for compensation. In addition, lower PI may decrease SS and flattened the lordosis; higher PI increases SS and lordosis is more pronounced. However, in our study LL was not different between the surgery group and the conservative group. The probable reason for this finding is that the fracture was in the thoracic spine in some patients, and some patients had multiple fractures. When measuring LL, the angle between the upper endplate of L1 and the upper endplate of S1 was recorded. Consequently, a vertebral compression fracture in lumbar spine would cause loss of LL, and if the fracture was in the thoracic spine there would be a compensatory increase of LL. Therefore, we measured the local kyphosis angle to represent spinal alignment change and local vertebral damage after vertebral compression fractures. From our statistical results, increased local kyphotic angle was significantly correlated to the need for surgical treatment (univariate logistic regression, *p* = 0.027, Table 2). Studies by Xie and Jin [35,36] indicated that an increased local kyphosis angle means there is more serious structural damage to the vertebrae and poorer stability, which leads to a higher risk of surgical intervention. 

Pain and disability are well-known clinical factors that predispose patients to receive surgical intervention [37]. In our study, patients in the surgery group had significantly higher vacuum clefts of fractured vertebrae, preoperative VAS pain scores, and ODI scores (Table 1). Vacuum clefts are radiolucent liner shadows representing subchondral bone necrosis due to insufficient revascularization during the bone healing process after vertebral fractures. There are two major reasons for pain and disability after a vertebral compression fracture. One is biological factors, including fracture nonunion, delayed union, and vertebral refracture. The other is mechanical factors, which are spinopelvic malalignment, local kyphosis, and postfracture instability [38]. Global spinal malalignment and poor spinopelvic parameters have been shown to affect the union of fractured vertebra [14]. Larger SVA and DSVA will cause the gravity center of the trunk to be farther from the “cone of economy”, and increase stress on the fractured vertebrae, leading to insufficient bone healing and avascular necrosis in the fracture site [39]. In our multivariate logistic regression analysis, we found that PT ≥ 27°, DSVA ≥ 6 cm, and PI not in the range of 44° to 62° predicted the need for surgical intervention with high accuracy (sensitivity = 0.708, specificity = 0.815, Figure 4). After controlling all variables, this model best predicted the probability of surgery.

Our study has some limitations. First, this study was nonrandomized and had a relatively short follow-up period. Due to the nature of observational cross-sectional cohort studies, there would be no significant difference in randomization. In this study, we focused on the impact of preoperative demographic data, global spinal alignment, and spinopelvic parameters, as related to the risk of surgical treatment. Therefore, a short follow-up period should not affect the conclusions. Second, there was some intraobserver bias in the measurement of spinopelvic parameters and global spinal alignment due to the influence of the fracture itself. Lastly, this study was from a single medical center. However, this study still established several cut-off points for predicting the outcomes of conservative management of patients with OVCFs. To confirm the results, randomized controlled trails with a larger number of patients are warranted in the future.

## 5. Conclusions

Patients with balanced spinopelvic parameters and sagittal alignment are more likely to respond to conservative management and not require surgery after OVCFs. This study found that SVA ≥ 50 mm, DSVA ≥ 60 mm, PI outside of the range of 44 to 62°, and PT ≥ 27° were associated with failure of conservative management and the need for surgical intervention. These results may help guide treatment of patients with OVCFs in acute or subacute stages.

## Figures and Tables

**Table 1:** Patient characteristics.

	Surgery (*n* = 25)	Conservative (*n* = 54)	*p*
Age (years)	73.28 ± 9.78	73 ± 8.58	0.420
Sex			0.138
Male	6 (24)	6 (11.11)	
Female	19 (76)	48 (88.89)	
BMD (T-score)	−3.28 ± 2.29	−2.71 ± 2.09	0.574
BMI (kg/m^2^)	24.33 ± 5.5	24.85 ± 3.28	0.746
Number of fractures			0.930
Single	22 (88)	47 (88.68)	
Multiple	3 (12)	6 (11.32)	
Pre-existing fracture			0.157
Yes	19 (35.19)	13 (52)	
No	35 (64.81)	12 (48)	
Fracture level			0.281
At TL	22 (88)	42 (77.78)	
non-TL	3 (12)	12 (22.22)	
Vacuum clefts			0.005 *
−	18 (72)	51 (94.4)	
+	7 (28)	3 (5.56)	
VAS (1st visit)	7 ± 0.89	6.47 ± 1.78	0.110
VAS (8 weeks)	5.39 ± 1.87	3.29 ± 1.59	<0.001 *
ODI (1st visit)	28.43 ± 6.18	25.84 ± 9.28	0.251
ODI (8 weeks)	21.83 ± 6.9	14.02 ± 7.68	<0.001 *

* Statistically significant. Data are presented as mean ± standard deviation, or number (percentage). BMD: bone mineral density; BMI: body mass index; TL: T10 to L2; VAS: visual analog scale; ODI: Oswestry disability index.

**Table 2:** Comparison of spinopelvic parameters and univariate regression.

	Surgery (*n* = 25)	Conservative (*n* = 54)	*p*	
			*t*-Test	Regression
Sagittal vertical axis (SVA) (mm)	71.89 ± 39.89	53.73 ± 49.6	0.245	0.122
Sacral slope (SS) (°)	33.28 ± 8.69	34.35 ± 9.61	0.601	0.631
Pelvic tilt (PT) (°)	29.42 ± 11.72	22.33 ± 9.09	0.124	0.008 *
Pelvic incidence (PI) (°)	62.68 ± 15.35	56.76 ± 10.48	0.021 *	0.054
Lumbar lordosis (LL) (°)	35.8 ± 15.74	34.85 ± 21.54	0.095	0.842
DSVA (mm)	64.09 ± 44.09	49.27 ± 48.14	0.651	0.199
Local kyphotic angle (°)	17.54 ± 10.93	9.88 ± 14.59	0.124	0.027 *

* Statistically significant. DSVA: horizontal distance between center of fractured vertebra and C7 plumb line.

**Table 3:** Univariate logistic regression for cut-off points of spinopelvic parameters for the need for surgery.

	Surgery (*n* = 25)	Conservative (*n* = 54)	OR (95% CI)	*p*
SVA			3.09 (1.14–8.41)	0.027 *
<50 (mm)	8 (32)	32 (59.26)		
≥50 (mm)	17 (68)	2 (40.74)		
DSVA			3.31 (1.23–8.90)	0.018 *
<60 (mm)	11 (44)	39 (72.22)		
≥60 (mm)	14 (35)	15 (27.78)		
PT			5.61 (2.01–15.67)	0.001 *
<27 (°)	9 (36)	41 (75.93)		
≥27 (°)	16 (64)	13 (24.07)		
PI			4.73 (1.72–13.05)	0.003 *
In 44–62°	10 (40)	41 (75.93)		
Out of 44–62°	15 (60)	13 (24.07)		
SS			1.25 (0.46–3.42)	0.664
<30 (°)	8 (32)	20 (37.04)		
≥30 (°)	17 (68)	34 (62.96)		
LL			0.97 (0.37–2.55)	0.95
<42 (°)	15 (60)	32 (59.26)		
≥42 (°)	10 (40)	22 (40.74)		

* Statistically significant. Data are presented as number (percentage). SVA: sagittal vertical axis; DSVA: horizontal distance between fracture center to C7 plumb line; PT: pelvic tilt; PI: pelvic incidence; OR: odds ratio; CI: confidence interval.
